# Recent Investigations in Medical Aid in Dying: Awareness, Preferences, Policies, and Clinical Practice

**DOI:** 10.1093/geroni/igaf122.865

**Published:** 2025-12-31

**Authors:** Samuel Nemeth, Elissa Kozlov

## Abstract

Medical Aid in Dying (MAID) is an increasingly available, legal end-of-life (EOL) option for Americans who meet eligibility criteria (less than 6 months prognosis, aged 18+, have capacity to consent to end of life medical care). Data suggests that while many in the US are unaware of MAID laws, there is considerable interest in potentially pursuing MAID if diagnosed with a terminal illness in the future. However, empirical studies of MAID at the macro and micro levels remains scarce. To that end, this symposium presents a collection of new insights on MAID. Nemeth, using data from a cross-sectional online survey, examines the potential demographic, belief, and experiences predictors of preference for future MAID use. Kozlov expands this discussion by analyzing these predictors and their relationships to US and State level certainty of knowledge of MAID legality. Phillips and Kumar explore and analyze hospice polices on MAID throughout the US. In the first of two studies, Becker, using cross-sectional survey data, examines hospice clinician in-person presence during patient MAID usage. In the second study, Becker continues this line of work by examining the timing (e.g., during self-administration) of clinicians in the room during patient MAID usage. Dr. Kozlov (discussant) will provide insights into current MAID policies, implications of the present studies, and directions for future research. These studies advance our understanding of MAID in the US by using a variety of data sources and populations, highlighting the need for and importance of unbiased, rigorous, empirical MAID research. Hospice, Palliative and End-of-Life Care Interest Group Sponsored Symposium

